# A Case Report of Calciphylaxis

**DOI:** 10.21980/J8KW8V

**Published:** 2025-07-31

**Authors:** Kim Hoang, Tien Lu, Alex Dang, Danielle Matonis

**Affiliations:** * University of California, Irvine, School of Medicine, Irvine, CA; ^ University of California, Irvine Health, Department of Emergency Medicine, Orange, CA

## Abstract

Calciphylaxis is a rare condition that is not well understood but is known to carry significant morbidity and mortality. We present a 44-year-old male with a history of end-stage renal disease on dialysis complaining of increasingly painful chronic wounds. The patient’s physical exam was remarkable for ulcerated, bilateral anterior leg wounds with large areas of eschar along with purulent drainage. He was admitted to the hospital for intravenous antibiotics, wound care, and specialist consultation for treatment of calciphylaxis. This case report provides an overview of the key clinical features of calciphylaxis and reviews potential treatment strategies for this life-threatening condition.

**Topics:**

Calciphylaxis, chronic kidney disease, end-stage renal disease, wound infection, wound care.

**Figure:**
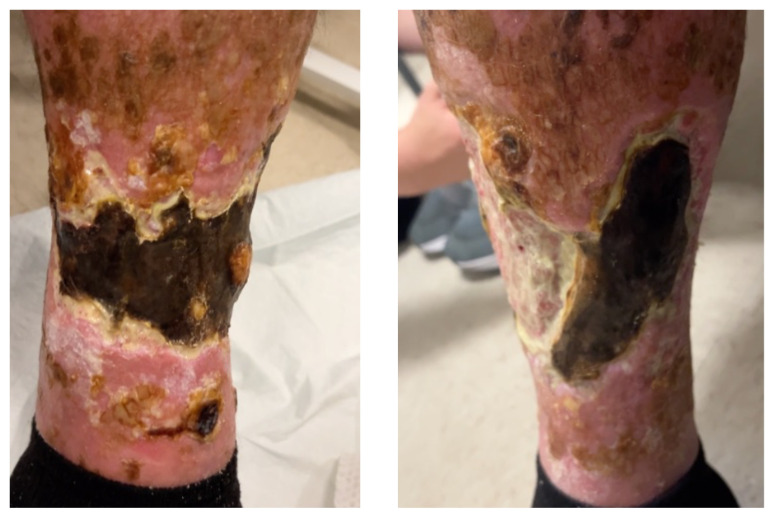
Video Links: https://youtu.be/3dkK9hj2hQs https://youtu.be/Gzl_h8ywQiM

## Brief introduction

Calciphylaxis is a painful condition characterized by the progressive calcification of arterioles resulting in poor-healing skin lesions that ulcerate, develop eschar, and undergo necrosis as the disease progresses.^1^ This condition most commonly affects patients on dialysis but can occur in patients with normal kidney function or those with chronic kidney disease (CKD).^2^,^3^ The prognosis of this disease is poor with an estimated one-year survival rate after diagnosis being nearly 50%.^4^ It is, therefore, an important diagnosis to consider in the emergency department (ED) to ensure appropriate follow up in this high-risk patient population. In this report, we review the case of a patient with known calciphylaxis presenting with worsening wounds, pain, and drainage.

## Presenting concerns and clinical findings

A 44-year-old male with a history of ESRD on hemodialysis (HD), peripheral arterial disease, hypertension, and type 2 diabetes presented to the ED for worsening pain and drainage from chronic wounds of the bilateral lower extremities. Four months previously, he was admitted to the hospital for new ulcerations and drainage at the site of painful red plaques of the bilateral shins that were worsening despite multiple rounds of antibiotics. He was seen by multiple specialists including dermatology, nephrology, and vascular surgery who felt the wounds were most consistent with calciphylaxis. Given this clinical picture, the specialty teams did not feel tissue biopsy was necessary for diagnosis. He was discharged with the plan for continued HD with the new addition of intravenous (IV) thiosulfate after each session as well as specific instructions regarding wound care and follow-up with our wound team.

The patient returned to the emergency room a few months later after being lost to follow-up. Despite attempts at wound care at home, his wounds continued to enlarge and become more painful. He reported general compliance with dialysis but that he may have missed a few sessions. Dialysis records were not available to confirm if or when he received IV thiosulfate. Over the preceding week, the patient noticed increased purulent drainage, worsening odor and was having chills at night, prompting his visit to the ED.

## Significant findings

On arrival for this visit, the patient was nontoxic appearing with stable vital signs. The physical exam was notable for deep, ulcerated, bilateral anterior leg wounds with purulent drainage and large areas of eschar (see photographs).

The labs obtained were notable for a sodium of 123 mEq/L, glucose of 483 mg/dL, anion gap of 15, creatinine of 7.1 mg/dL, GFR of 8, and no leukocytosis. Worsening peripheral arterial disease was considered as a possible contributor to the worsening disease progression and poor wound healing. A computed tomography (CT) angiogram of the abdomen and pelvis with bilateral lower extremity runoff was obtained and showed extensive calcified plaques in several arteries throughout the bilateral lower extremities. While notable findings, these calcifications and stenoses were unchanged from prior imaging.

## Patient course

The patient was started on IV vancomycin and ceftriaxone while in the ED. Given the poor wound healing and arterial stenoses on imaging, vascular surgery was consulted. They agreed with starting IV antibiotics and admitting to medicine for further evaluation and management. After reviewing the imaging and evaluating the patient, vascular surgery ultimately determined that vascular intervention was not indicated and was unlikely to alter the clinical course. They recommended wound care management and considering burn surgery consultation for potential debridement. Burn surgery agreed with vascular surgery’s recommendation for wound care management but recommended against debridement. The patient received hemodialysis during the hospital stay, along with sodium thiosulfate on days of hemodialysis sessions per nephrology recommendations. Eventually, the patient was discharged with oral antibiotics, modifications to his insulin regimen for better glucose control, sodium thiosulfate to be given after every dialysis session, and referred to wound clinic for continued management of calciphylaxis and chronic wounds.

## Discussion

Calciphylaxis is an increasingly prevalent condition associated with high morbidity—including pain, impaired wound healing, and frequent hospitalizations—and high mortality, most often due to sepsis.^1^ Calciphylaxis disproportionately affects patients with ESRD, although it may also occur in patients with any other level of kidney function.^1^–^3^ Our patient demonstrated risk factors for calciphylaxis including uncontrolled diabetes mellitus and a history of ESRD on dialysis.^1^,^2^ Additional risk factors for this condition include comorbidities such as obesity, dysregulated calcium-phosphorous metabolism, and certain medications.^1^,^5^

While the pathophysiology of calciphylaxis is not completely understood, it is hypothesized that a combination of vascular injury and vessel calcification plays an important role in the early development of calciphylaxis plaques.^1^,^6^ This hypothesis is based on the finding of vessel calcification found in all histological tissue samples from calciphylaxis patients.^6^ Additionally, uremic patients are known to have dysregulated calcium and phosphate homeostasis which is presumed to contribute to the calcification process.^1^,^4^ The resulting vascular occlusions cause poor wound healing and increase the risk of ulcerations with superimposed infection.^1^

Clinical presentation of calciphylaxis primarily manifests as painful, poor-healing cutaneous lesions surrounded by reticulate purpura and firm calcified subcutaneous tissue.^1^,^4^ These lesions often form ulcerations that can rapidly develop necrotic eschar as vascular thrombosis progresses.^4^,^7^ Subcutaneous plaques most commonly develop on the lower extremities, although lesions can be found elsewhere, including the abdominal wall and upper extremities.^4^ Calciphylaxis in our patient was suspected due to a history of ESRD with the physical presentation of chronic, painful, non-healing wounds. It is important to highlight that several other diseases may present with similar dermatologic findings that resemble calciphylaxis such as peripheral vascular disease, atherosclerotic disease, warfarin-associated necrosis, and cryoprecipitate disorders.^1^,^7^,^8^

Given that the primary manifestations of calciphylaxis are dermatologic, initial evaluation involves assessing for painful, cutaneous nodules.^4^,^7^,^9^ While a skin biopsy is the most optimal way to rule out other conditions, the risk-to-benefit ratio for patients remains unclear given the risk of further ulceration and infection.^8^,^9^ There is a lack of consensus regarding the diagnostic utility of laboratory evaluation. Some studies have suggested its use to assess potential risk factors such as renal function, liver function, bone mineral parameters, and coagulation profile.^1^,^9^ However, while disturbances in phosphate and calcium levels serve as risk factors for calciphylaxis, not all patients present with these findings, thereby limiting the utility of laboratory testing.^10^ Since biopsies are unlikely to be performed in the ED and laboratory values are nonspecific, an official diagnosis may not be made during an ED visit, but a high index of suspicion is crucial in identifying patients with potential or likely calciphylaxis.

While there is no FDA-approved treatment, current medical management focuses on preventing further vascular calcification through various mechanisms^10^. Mineral imbalances such as hypercalcemia and hyperphosphatemia are addressed via dietary modifications and by giving medications that increase calcium and phosphate clearance.^1^,^8^,^10^ For example, sodium thiosulfate is thought to increase the solubility of calcium to aid in its eventual clearance and is often given after each round of dialysis.^1^,^10^ Medications such as cinacalcet that reduce parathyroid hormone and the consequent release of calcium can be used to treat hypercalcemia indirectly.^1^,^10^ Other treatment includes discontinuing medications that serve as risk factors for calciphylaxis, such as calcium supplements, warfarin and corticosteroids.^5^

Proper wound management is another crucial component of calciphylaxis care.^10^ Management of the wound bed should prioritize moisture control and respective wound care teams should be consulted for specific dressing recommendations.^1^,^10^ Literature has demonstrated successful ulcer healing after surgical debridement with conventional wound dressings such as paraffin gauze and hydrocolloids.^11^ Surgical debridement itself remains controversial. Debridement of large wounds or wounds with necrotizing tissue is recommended for the prevention of further infection; however, there remains the risk of wound enlargement and continued poor healing.^10^,^11^ One small single-center study demonstrated that patients with calciphylaxis who underwent debridement versus those that did not had a survival rate of 61.6% and 27.4%, respectively.^4^ It is important to note that this study is limited by the lack of control between patients. However, experts still debate the specific indications and technique for debridement and in which patients it may best help with wound healing.^10^ Several other therapies have been trialed over the years including hyperbaric oxygen therapy, but there has been a lack of clear positive outcomes.^4^,^10^

No consensus currently exists for the use of antibiotics for wounds secondary to calciphylaxis. A multi-interventional treatment strategy for calciphylaxis at a single institution showed support for the use of antibiotics while other literature has shown no indication for the prophylactic use of antibiotics.^12^,^13^ The literature does not provide specific antibiotic recommendations, but it is assumed that antimicrobial therapy must be individualized in the presence of infection given that sepsis is the leading cause of death in this patient population.

In summary, while the workup, definitive diagnosis, and treatment of calciphylaxis may not occur in the emergency department, it is still important for emergency physicians to consider this diagnosis in their differentials and understand the basics of treatment since it carries a significant degree of morbidity and mortality. In a patient with worsening or poorly healing wounds with risk factors of calciphylaxis, referring the patient for close multispecialty follow-up, or even admitting them in severe cases, can aid in reaching an appropriate diagnosis and treatment plan for this complex disease.

## Supplementary Information








