# Supplementary figures and images for: A Case Report of Calciphylaxis
Source: J Educ Teach Emerg Med. 2025 Jul 31;10(3):V27–30. doi: 10.21980/J8KW8V (PMC12320990; doi:10.21980/J8KW8V)

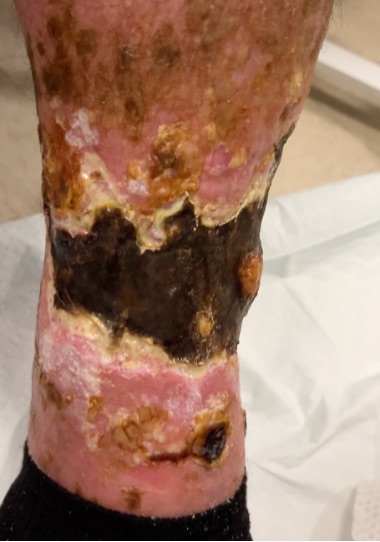

Supplement: Supplementary file 1 [file 10-3-V27-Supp1.jpeg]

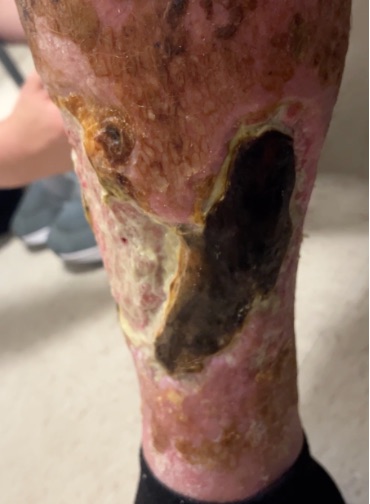

Supplement: Supplementary file 2 [file 10-3-V27-Supp2.jpeg]
